# Geotechnical Characteristics and Stability Analysis of Rock-Soil Aggregate Slope at the Gushui Hydropower Station, Southwest China

**DOI:** 10.1155/2013/540636

**Published:** 2013-09-02

**Authors:** Jia-wen Zhou, Chong Shi, Fu-gang Xu

**Affiliations:** ^1^State Key Laboratory of Hydraulics and Mountain River Engineering, Sichuan University, Chengdu, Sichuan 610065, China; ^2^State Key Laboratory of Geohazard Prevention and Geoenvironment Protection, Chengdu University of Technology, Chengdu, Sichuan 610059, China; ^3^Research Institute of Geotechnical Engineering, Hohai University, Nanjing, Jiangsu 210098, China; ^4^College of Water Resources & Hydropower, Sichuan University, Chengdu, Sichuan 610065, China

## Abstract

Two important features of the high slopes at Gushui Hydropower Station are layered accumulations (rock-soil aggregate) and multilevel toppling failures of plate rock masses; the Gendakan slope is selected for case study in this paper. Geological processes of the layered accumulation of rock and soil particles are carried out by the movement of water flow; the main reasons for the toppling failure of plate rock masses are the increasing weight of the upper rock-soil aggregate and mountain erosion by river water. Indoor triaxial compression test results show that, the cohesion and friction angle of the rock-soil aggregate decreased with the increasing water content; the cohesion and the friction angle for natural rock-soil aggregate are 57.7 kPa and 31.3° and 26.1 kPa and 29.1° for saturated rock-soil aggregate, respectively. The deformation and failure mechanism of the rock-soil aggregate slope is a progressive process, and local landslides will occur step by step. Three-dimensional limit equilibrium analysis results show that the minimum safety factor of Gendakan slope is 0.953 when the rock-soil aggregate is saturated, and small scale of landslide will happen at the lower slope.

## 1. Introduction

Rock-soil aggregate is widely distributed in China and worldwide [1, 2]. At present, numerous hydropower stations are being planned, constructed, or operated in Southwest China. Rock-soil aggregate creates a difficult problem, especially in the mountainous region near the Tibet Plateau. The formation history of rock-soil aggregate is very complicated. Rock-soil aggregates are mainly a result of slope deposits, colluvial, alluvial, and fluvial deposits. The material composition of rock-soil aggregate is also complex; its structure distribution is extremely irregular, and geographic and other characteristics add to the complexity [3–5]. The mechanical characteristics of rock-soil aggregate are between soil and rock. Second, the rock-soil aggregate is formed in the stage of mountains eroded by river, which is the active period of slope deformation and failure [6–8]. Furthermore, in the geological evolution process of slopes, rock-soil aggregate supply is controlled by the interaction of climate and tectonic uplift/subsidence [9, 10].

The existence of large rock-soil aggregate slopes has a tremendous impact on projects, such as the stability of the slope for a reservoir storing water. Landslides, debris flows and other disasters are often caused by rock-soil aggregate slope under rainfall and earthquake conditions [11]. The relationship between landslides and rock-soil aggregate delivery has a serious impact on dam safety because some sliding of rock-soil aggregate slopes can reach the river channel [12]. Landslides of rock-soil aggregate slopes are often caused by heavy rainfall conditions. For the rock-soil aggregate slopes, the geological processes, status evaluation, mechanical characteristics, and stability evaluation are the key issues. However, due to the complex mechanical properties of rock-soil aggregate slopes, it is difficult to make an accurate analysis of the related rock-soil aggregate problems [13].

Rock-soil aggregate is generally considered a highly complex discontinuous material [14]. Because the mechanical characteristics of rock and soil are different and the interactions of rock soil are complicated, the mechanical characteristics of rock-soil aggregate are unlike a homogeneous material. Instead, the characteristics depend on the particle size distribution and the microstructure characteristics [15, 16]. The shear strength is a key mechanical characteristic of rock-soil aggregate related to the slope stability evaluation, and the cohesion and friction angle are usually used to describe the shear strength of the rock-soil aggregate [17]. The cohesion and friction angle of rock-soil aggregate are influenced by the particle size distribution characteristics and other conditions, especially the water content.

A large volume rock-soil aggregate slope (Gendakan slope) at the Gushui Hydropower Station is selected for case study in this paper. The geological evolution process of the rock-soil aggregate slope is analysed. Combined with a field geological survey and experimental tests based on the geological process analysis of the rock-soil aggregate slope, the layered characteristics of rock-soil aggregate and the toppling failure of plate rock masses are presented. The physical characteristics and particle size distribution of the rock-soil aggregate at the Gushui Hydropower Station are analysed. Field tests of shear strength and the relationship of cohesion, friction, and water content are presented. Finally, the deformation and failure mechanism of the rock-soil aggregate slope is analysed, and a three-dimensional limit equilibrium method is adopted to compute the safety factor of slope.

## 2. Geological Setting

**Figure 1:**
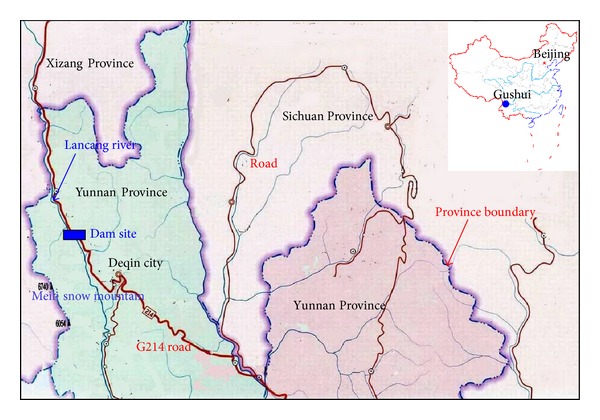
Site location of the Gushui Hydropower Station.

The Gushui Hydropower Station is located at the upper stream of the Lancang River, Xingzheng Village, Foshan Town, Northwest of Deqin City, Yunnan Province.  Figure 1 shows the site location of the Gushui Hydropower Station. The drainage area of the Gushui Hydropower Station is 8.35 × 10^4^ km^2^, the water level elevation of the reservoir is 2,340 m, the dam type is rockfill, with a height of 300 m, and the storage capacity of the water is 39.12 × 10^8^ m^3^. The power installation of Gushui Hydropower Station is 2,600 MW, and the annual average power generation is 109.9 × 10^8^ kWh.

### 2.1. Rock-Soil Aggregate

Different types of accumulations are distributed in the project site region, including alluvium accumulation, talus accumulation, collapse accumulation, and outwash accumulation. The main type is outwash accumulation because of the rock and soil particles carried by glacier melt water. The accumulation can be divided into 4 types according to the deposit process: fluvial deposits, flood deposits, collapse or residual deposits, and outwash deposits.

**Figure 2:**
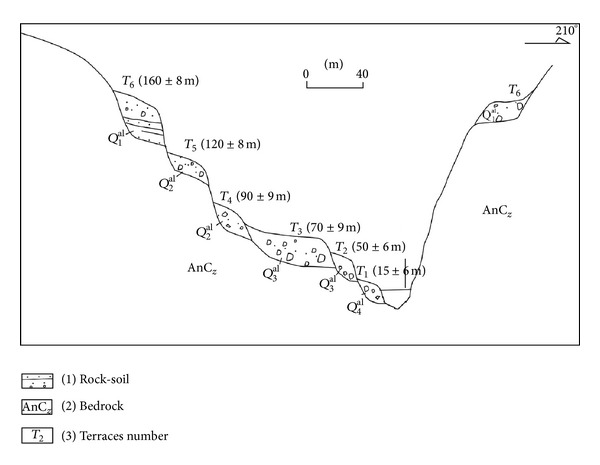
Typical fluvial deposit terrace of the Lancang River in the Gushui Hydropower Station region.

1. Fluvial deposits: Figure 2 shows the typical fluvial deposit terraces section of the Lancang River. Because the mountain is influenced by the new tectonic movement, the mountain valley erosion phenomenon is obvious. The fluvial deposits are distributed continuously along the Lancang River bank. In the dam site region, 5-6 terraces existed as fluvial deposits, as shown in Figure 2. *T*_1_ is the Holocene terrace, *T*_2_ and *T*_3_ are the late Pleistocene terraces, *T*_4_ and *T*_5_ are the middle Pleistocene terraces, and *T*_6_ is early Pleistocene terrace.
2. Flood deposits: the flood deposits located at the export of a valley or gully are distributed like a fan because of the rock and soil particles carried by the flood after the late Pleistocene. The rock-soil aggregate is composed of gravel, sand, and rock blocks. The scale of flood deposits is small, and the particle sorting is general.
3. Collapse or residual deposits: the collapse or residual deposits are distributed at the foothills of the Lancang River. The main compositions are the deposits of rockfall or the toppling failure of slopes.
4. Outwash deposits: outwash deposits are the main type of the rock-soil aggregate slope in this dam site region. The rock and soil particles carried by the glacier melt water have left many traces that record the outwash deposit process. Below an elevation of 3,000 m, the slope is mostly covered by rock-soil aggregate.

### 2.2. Bedrock

**Figure 3:**
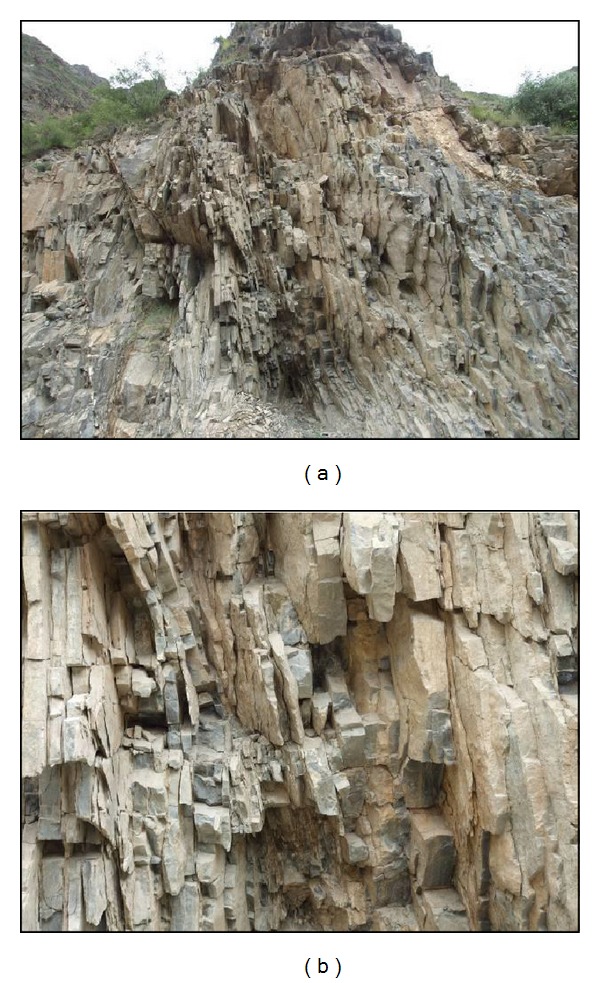
Rock masses in the Gushui Hydropower Station region: (a) and (b) are the exposed plate sandstone.

The main rock types are sandstone, mudstone, limestone, and basalt. The rock mass is strong and hard. The typical characteristic is that the rock layers are close to vertical, the bedrock is mostly plate rock masses, and toppling failure occurs in the plate rock masses.  Figure 3 shows the plate rock masses in the Gushui Hydropower Station region.

At the left bank of the project site region, the strong weathering depth of rock masses is less than 30 m, the horizontal depth of the weak weathering is approximately 100 m–200 m, and vertical depth is also approximately 100 m–200 m. At the right bank of the dam site region, the strong weathering depth of rock masses is less than 50 m, the horizontal depth of weak weathering is approximately 80 m–280 m, and the vertical depth is approximately 70 m–250 m. The weathering degree of rock masses is asymmetric between the left and right banks. The unloading depth of the rock masses at the left bank is approximately 30 m–50 m, and the right bank is approximately 50 m–100 m.

## 3. Geological Analysis of Layered Accumulations and Toppling Plate Bedrock

### 3.1. Rock-Soil Aggregate Slope at the Gushui Hydropower Station Region

**Figure 4:**
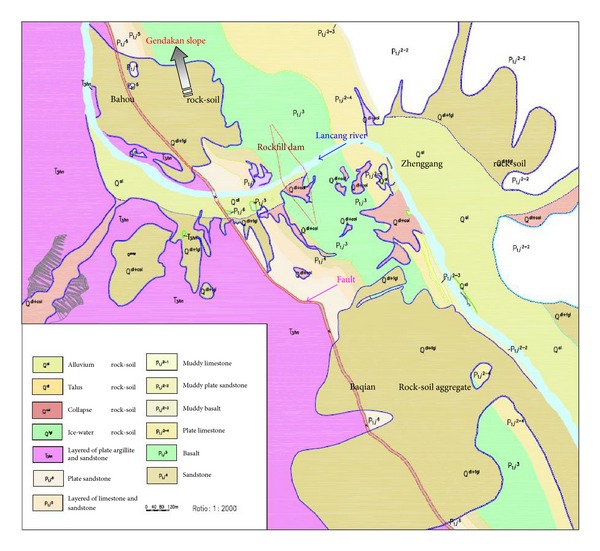
Rock-soil aggregate slope distribution in the dam site region of the Gushui Hydropower Station.

At the Gushui Hydropower Station region, numerous rock-soil aggregate slopes are distributed at both sides of the river valley.  Figure 4 shows the rock-soil aggregate slope distribution in the project site of the Gushui Hydropower Station.

**Figure 5:**
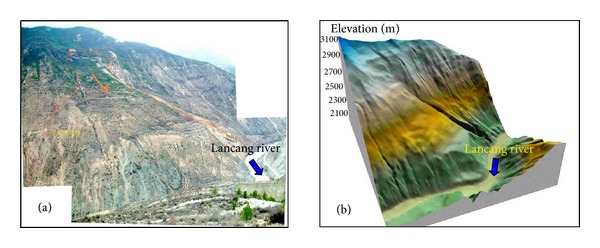
The Gendakan slope at the Gushui Hydropower Station: (a) photograph of the Gendakan slope; (b) three-dimensional visualization.

As shown in Figure 4, there are numerous rock-soil aggregate slopes at different elevations. The slope scale ranges from small to extremely large, and the volume of each rock-soil aggregate slope and its impact on the hydropower station are different. In the dam site region of the Gushui Hydropower Station, the safety of the station at the construction and operation stages is influenced by 4 very large rock-soil aggregate slopes: the Gendakan slope, the Bahou slope, the Baqian slope, and the Zhenggang slope. In this paper, the Gendakan slope is selected as an example for study.  Figure 5 shows the Gendakan slope at the Gushui Hydropower Station, located at the middle of reservoir, is approximately 4 km away from the dam site and is distributed at an elevation of 2,060 m–2,800 m. The terrain slope is approximately 20°–30°, and there are three tablelands at elevation of 2,550 m, 2,400 m, and 2,250 m. The Gendakan slope is mainly composed of outwash deposits with a layered structure, and the main particles are rock block, broken stone, and silt. The thickness of the outwash accumulation is approximately 70 m–80 m, the maximum thickness is approximately 230 m, and the volume is greater than 3,000 × 10^4^ m^3^.

### 3.2. Layered Characteristics of Outwash Accumulation

**Figure 6:**
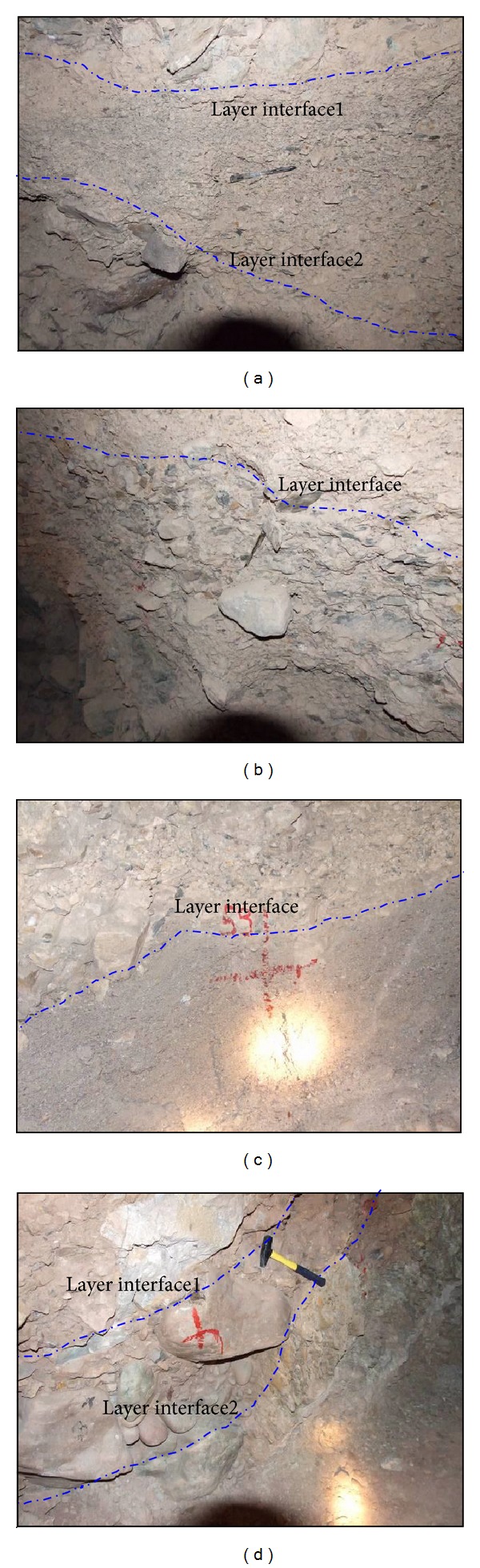
Layered rock-soil aggregate in the PD 33: (a) and (c) are the small size particle rock-soil aggregates; (b) and (d) are the large size particle rock-soil aggregates.

In the Gushui Hydropower Station region, the outwash accumulation is well developed below 4,000 m, especially below the 3,000 m. The main reason for the formation of the rock-soil aggregate is the melting of the glaciers, which generates surface water. An enormous amount of rock and soil particles are carried by the outwash, and they flow downward and are deposited. The rock-soil aggregate is mostly composed of rock block, broken stone, and clay or sandy soil. Because the geological history of the rock-soil aggregate formation is long, the slope evolution can be divided into many stages, and the layered characteristic of outwash accumulation is obvious.  Figure 6 shows the layered rock-soil aggregate in the PD 33 (PD is a horizontal exploration tunnel).

As shown in Figure 6, the particle size of each rock-soil aggregate layer is different, and there exist nonuniform distribution characteristics. In a special deposit stage, the rock-soil aggregate will be composed of small particles of rock block, sand, and soil, as shown in the middle rock-soil aggregate layer of Figure 6(a) and the lower rock-soil aggregate layer of Figure 6(c). The average particle size is approximately 15–25 mm. In another special deposit stage, the rock-soil aggregate is composed of large particles of boulders, big rock blocks, and sandy soil, as shown in the lower rock-soil aggregate layer of Figure 6(b) and the middle rock-soil aggregate layer of Figure 6(d). The average particle size is approximately 80–150 mm. The size ratio of the small particles in Figure 6(b) and the big particle in Figure 6(a) is approximately 5–10, and it will be larger in another condition. The particle size of one rock-soil aggregate layer is dependent on the carrying capacity of outwash or rainfall. When the intensity of glacier melting and rainfall is heavy in a special historical stage, the particle size will be large, but in another rock and soil deposit history stage, the particles will be small [18].

Combined with a field geological survey and an experimental test of the rock-soil aggregate, other physical characteristics of the rock-soil aggregate are as follows.

1. The rock-soil aggregate can be simplified as a two-phase structure: soft clay and hard rock block. Soft clay is the main component, and hard rock block is the filling material. The range of the particle size is large.
2. The different particle sizes of rock block are distributed in the soft clay randomly, exhibiting inhomogeneity and randomness. In the deposit process, the particle sizes are influenced by the terrain and the carrying capacity of the water flow. The rock block content will be very large in some regions, but in other regions the rock block content will be small.

### 3.3. Toppling Failure of Plate Bedrock

**Figure 7:**
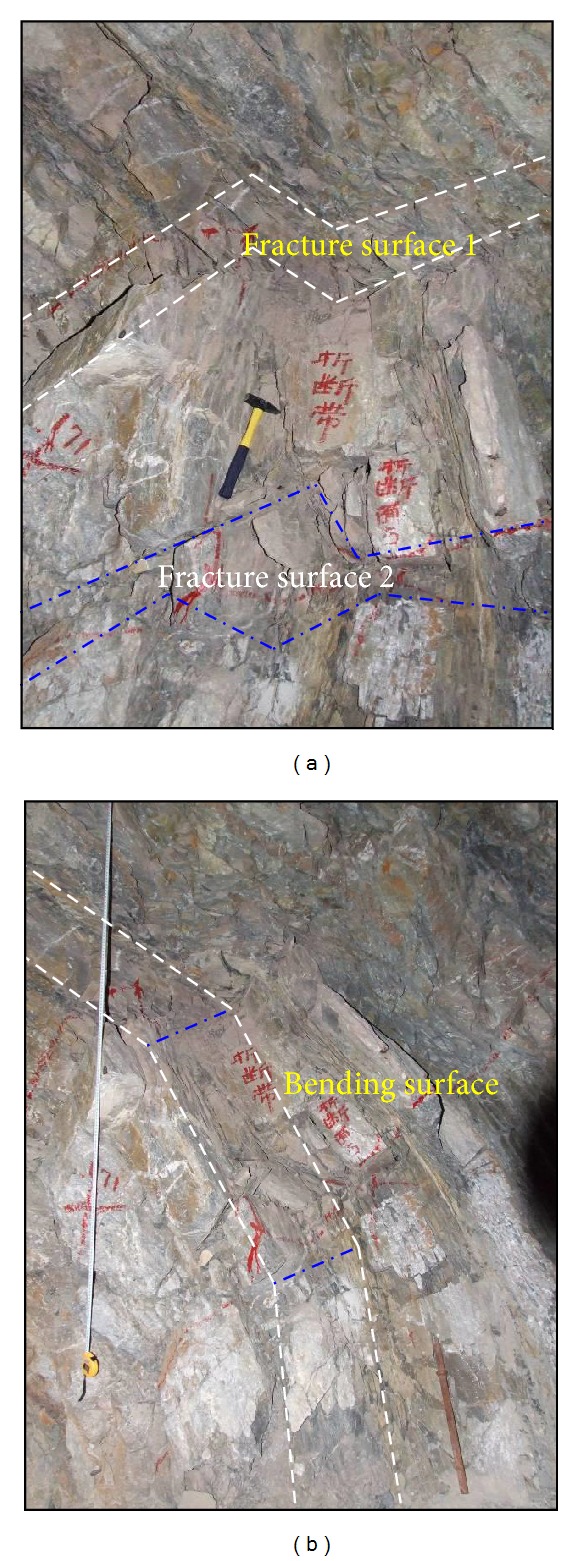
Toppling failure of plate rock masses in the PD 13: (a) the bending fracture surface in the horizontal direction and (b) the bending fracture surface in the vertical direction.

In the Gushui Hydropower Station region, the bedrock is most of the plate rock masses, and the rock layers are thick. There are mainly plate sandstone, plate limestone, and plate basalt, and the rock layers are nearly vertical. In the deep valley region, the plate rock masses are influenced by the weight of the upper rock-soil aggregate and gravity itself. The failure occurs in the rock block, and the toppling failure occurs for the plate rock masses along the slope direction. The toppling failure of plate rock masses only occurs at a certain depth, not in the deepest parts of the slope. The erosion of the river valley and the increase of the upper rock-soil aggregate weight and thickness of the plate rock layer are the main reasons for the toppling failure of plate rock masses [19]. The toppling failure phenomenon is extremely common in this region and has a great impact on the Gushui Hydropower Station.  Figure 7 shows the toppling failure phenomenon of plate rock masses in the PD 13. The fracture of rock and bending of plate rock mass exist in the toppling failure rock masses.

Based on the field geological survey of plate rock masses, two main physical characteristics of the toppling failure of plate rock masses are as follows.

1. Rock mass structure characteristic: a longitudinal thin-bedded structure along the slope direction and an alternate layer of soft and hard rock mass;
2. Spatial distribution characteristic: in the vertical section, a discontinuity surface exists for rock layers, and the bending phenomenon of plate rock masses is apparent; in the horizontal section, the toppling failure of the plate rock masses is distributed at different spacing parallel to each other, and the shear dislocation phenomenon is common.

The toppling failure of plate rock masses can be divided into different stages, and the geological history is long. The toppling failure can be divided into two types: strong toppling and weak toppling. The classification of toppling failure is according to the angle of the toppling rock layer and the normal rock layer and the physical chrematistics of the failure surface.  Table 1 shows the classification of the toppling failure of plate rock masses.

As shown in Table 1, strong toppling is when the angle of the toppling rock layer and the normal rock layer is greater than 60°, while weak toppling is when the angle of the toppling rock layer and the normal rock layer is less than 60°. The strong toppling always occurs in the upper slope at the upper part of the plate rock masses, and the weak toppling is below the strong toppling. The strong toppling occurs at a horizontal depth of approximately 50 m, but the strong toppling is at approximately 100 m. 

A toppling failure example in the Baqian slope illustrates the physical characteristics of strong toppling and weak topping. The dip of the Baqian slope is approximately 20°–40°. The toppling failure occurs in the plate sandstone, limestone, and mudstone. The normal rock layer is oriented in the dip direction of 325°–335° and the dip of 75°–90°. The dip of the weak topping rock layer is 40°–50°, and the strong toppling is 20°–35°. The horizontal depth of strong toppling is approximately 29.1 m–72.5 m, and weak toppling is approximately 90.7 m–111.2 m. The fracture and bending of the rock block and the dislocation of the rock layers result in the toppling of plate rock masses.

### 3.4. Geological Process of the Slope

**Figure 8:**
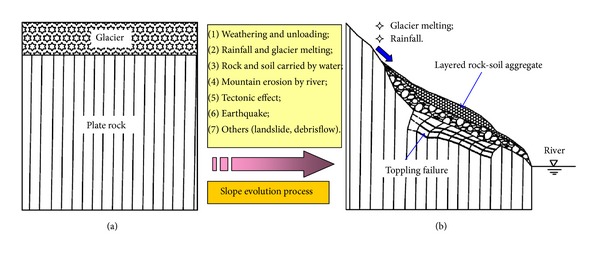
Geological evolution process of slope in the Gushui Hydropower Station region: (a) ancient landscape and (b) current landscape.

Based on the above geological analysis of rock-soil aggregate and toppling failure, the key characteristic of rock-soil aggregate is that it is layered, and for the toppling failure of plate rock masses, there are several fracture surfaces.  Figure 8 shows the geological evolution process of slopes in the Gushui Hydropower Station region.

As shown in Figure 8, the geological evolution process of slopes is influenced by several factors, including weathering and unloading of rock masses, glacier melting, rainfall, rock and soil particles carried by water flow, mountain erosion by rivers, tectonic effects, earthquakes, and other factors. In the ancient glacier geological stage, plate rock masses are covered by a very thick glacier, and the dips of the rock layers have a nearly vertical orientation, as shown in Figure 8(a). Along with global climate change and the influence of rainfall and tectonic movements, the mountain is also eroded by river water. In this process, the glacier melts and ice water are generated, and the amounts of rock and soil particles at the slope surface are carried by the movement of ice water; they migrate to the lower part of the slope, and the depth of the rock-soil aggregate increases gradually. In the geological evolution process, the rock-soil aggregate generated by the ice water gradually increased at various stages, resulting in a layered effect. The melting of glaciers and the weathering of rock masses in shallow slopes are two key factors for the rock-soil aggregate.

For the plate rock masses in the long geological evolution history, first, the deformation and strength parameters of the rock masses are decreased by the weathering and unloading effects. Second, the water flow will also impact the plate rock masses. The failure of the rock masses will occur, but these effects are not the main reasons for the toppling failure of the plate rock masses. The key reasons for the toppling failure of plate rock masses are the increasing weight of the upper rock-soil aggregate and the mountain erosion by river water. Also, the stress in the shallow plate rock masses is increased. Combined with the increasing stress and decreasing rock mass mechanics parameters, toppling failure occurs. The fracture of rock block and the dislocation of rock layers are also influenced by several geological stages. Because the weight of the upper rock-soil aggregate increases, the mountain is eroded by river water, and the weathering and unloading of rock masses are gradual processes. The result is several distinct stages in the toppling failure trace.

For the slope stability problem and its impact on the Gushui Hydropower Station, the likelihood of landslide in the rock-soil aggregate is greater than in the toppling failure plate rock masses, and the shallow landslide risk of the rock-soil aggregate is greater than the deep landslide along the joint surface.  Table 2 shows an example of shear strength test results for plate basalt and joint surface (triaxial compression experiments are carried out for basalt, and direct shear tests are carried out for joint surface).

As shown in Table 2, the shear strength parameters of basalt and joints are higher, and the landslide probability along the toppling plate rock masses is very low. The mechanical characteristics and slope stability analysis of rock-soil aggregate are the key issues in the following, and it is the focus of the engineer.

## 4. Geotechnical Characteristics of Rock-Soil Aggregate

In this section, the Gendakan slope is selected for the geotechnical characteristics analysis of rock-soil aggregate. Forty-seven test pits, 13 vertical boreholes and 7 horizontal geological tunnels were made for the geological survey work, and 50 indoor triaxial compression experiments (the test sample is a cylinder, diameter is 300 mm, and height is 600 mm) were performed to determine the mechanical characteristics of the rock-soil aggregate. First, the physical characteristics of the rock-soil aggregate are analysed based on the field survey and experimental test results; second, the particle size distribution characteristics are analysed; finally, the shear strength of the rock-soil aggregate influenced by the water content is analysed based on the experimental test results.

### 4.1. Physical Characteristics

**Figure 9:**
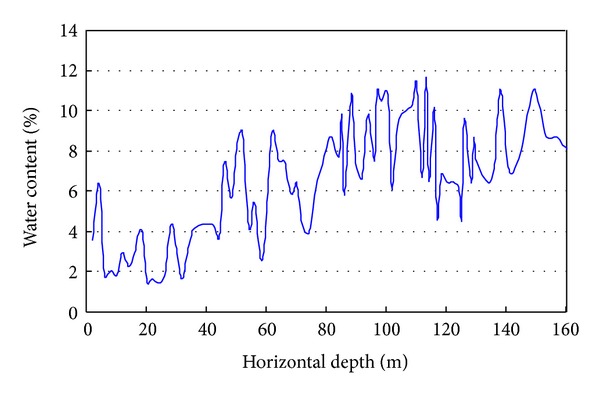
Water content of rock-soil aggregate varied with the horizontal depth.

The rock-soil aggregate is composed of several minerals: quartz and plagioclase are the main components; dolomite, calcite, and sericite are the secondary components; and chlorite and kaolinite are the minor components. The density of the rock-soil aggregate is approximately 1.95–2.21 g/cm^3^.  Figure 9 shows the water content of the rock-soil aggregate varied with the horizontal depth in PD 33.

As shown in Figure 9, the natural water content is increased with the horizontal depth. The maximum water content is 11.95%, the minimum water content is 1.76%, and the average water content is approximately 6.35%. The average water content of the rock-soil aggregate is greater than 6% when the horizontal depth is greater than 50 m. The water content in the shallow slope is less than the deep slope.

### 4.2. Particle Size Distribution

**Figure 10:**
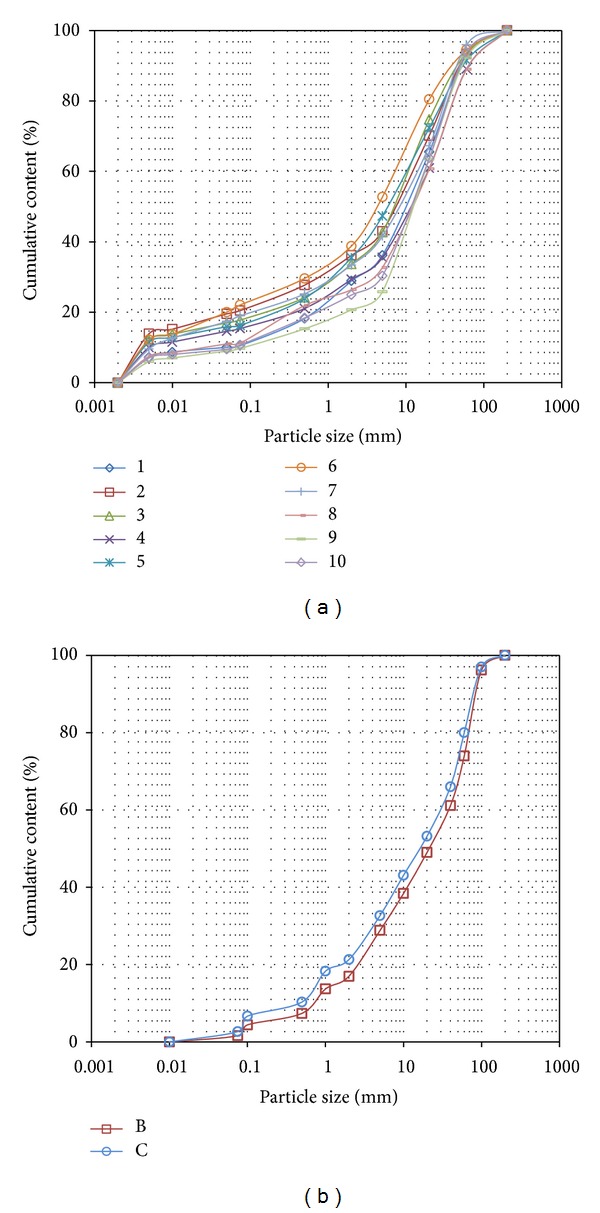
Statistical results for particle size distribution of rock-soil aggregate at the Gendakan slope: (a) field test results and (b) indoor test results.

A field particle screening test was conducted for the particle size distribution of rock-soil aggregate in the vertical depth of 5–10 m at the Gendakan slope for 10 groups.  Figure 10(a) shows the particle size distribution curves of rock-soil aggregate in the field. For the bottom sliding layer of rock-soil aggregate, the shear strength is crucial for the slope stability of the rock-soil aggregate, so two indoor experiments were conducted for the bottom rock-soil aggregate.  Figure 10(b) shows the particle size distribution curves of the bottom sliding layer of rock-soil aggregate.

The field test and indoor experiment results show that the rock-soil aggregate is composed of clay breccia, fine-grained soil, and rock block. The particle size distribution characteristics of the rock-soil aggregate are as follows.

1. The rock block content of a particle diameter less than 5 mm is approximately 32.46%, and the rock block content of a particle diameter greater than 5 mm is approximately 67.54%.
2. The rock block content of a particle diameter greater than 60 mm is approximately 7.34%.
3. The soil content of a particle diameter less than 0.075 mm is approximately 15.29%, and the soil content of a particle diameter less than 0.005 mm is approximately 9.7%.

### 4.3. Shear Strength

The mechanical characteristics of the rock-soil aggregate are sensitivity to the water content and shear strength decreasing with the increasing water content. The stability of the rock-soil aggregate slope is affected under rainfall or water conditions. For the rock-soil aggregate slope at the Gushui Hydropower Station, parts of the rock-soil aggregate slope will be under the water level when the reservoir is working, and the shear strength of the rock-soil aggregate will decrease and impact the slope stability. The rock-soil aggregate impounded for an extended period of time will cause the shear strength to decrease further. Furthermore, the shear strength of rock-soil aggregate under heavy rainfall or high water level conditions is influenced by the microstructure of the rock-soil aggregate, the content of rock block, the particle size distribution characteristics, and other factors. Therefore, the shear strength of rock-soil aggregate under water is highly complex.  Table 3 shows some cohesion and friction values for rock-soil aggregates in China.

**Figure 11:**
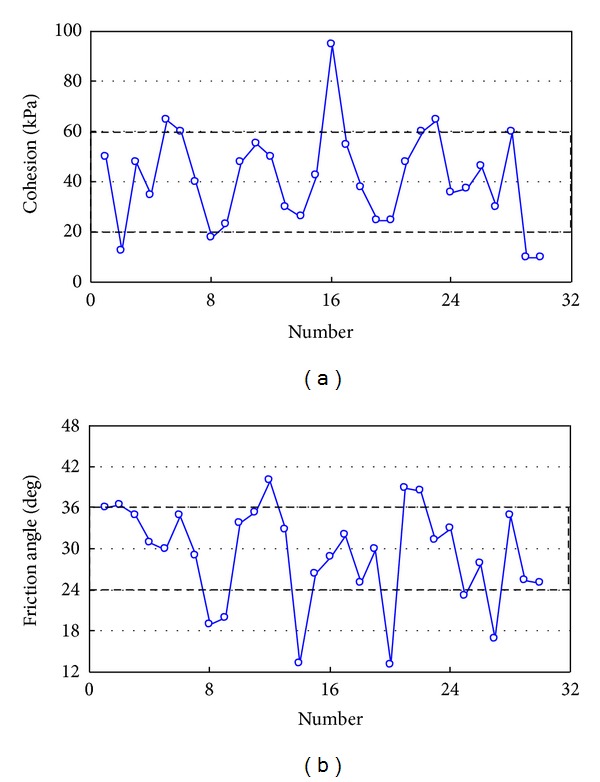
Statistical results for shear strength of rock-soil aggregate in China: (a) cohesion and (b) friction angle.

As shown in Table 3, the shear strength of cohesion is influenced by the material composition, particle size distribution characteristics, and water content, and obviously the shear strength is not the same for different rock-soil aggregates. For the shear strength of rock-soil aggregates, however, there is a certain range of cohesion and friction angle.  Figure 11 shows the statistical results for shear strength of rock-soil aggregates in China.

As shown in Figure 11, the statistical results are 30 sets of shear strength of rock-soil aggregate. Most cohesion values are in the range of 20–60 kPa, and most friction angles are in the range of 24°–36°.

**Figure 12:**
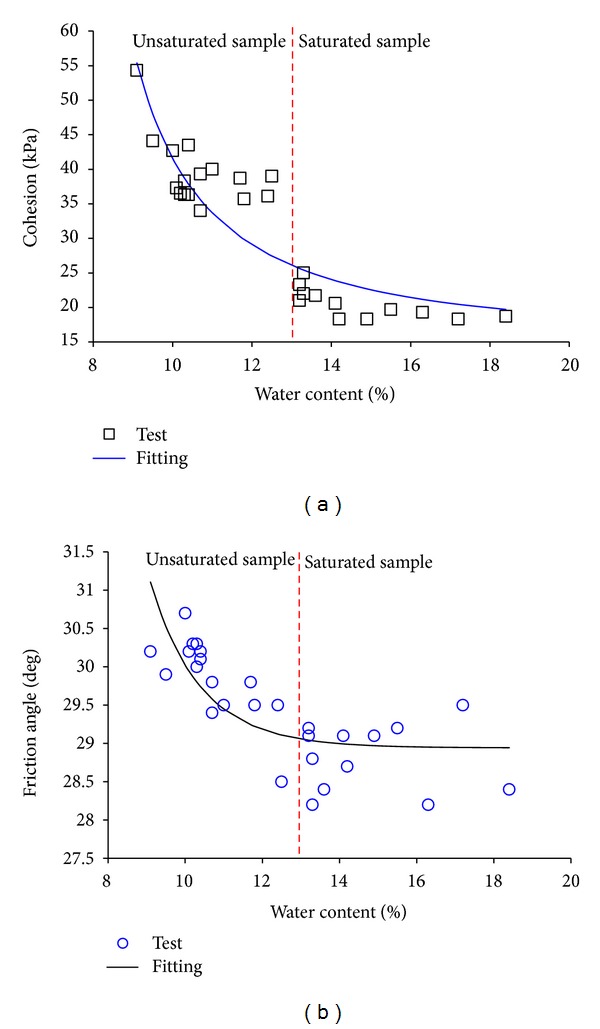
Shear strength test results of rock-soil aggregate under different water content conditions: (a) cohesion and (b) friction angle.

Fifty triaxial compression experimental tests were performed to determine the shear strength of the rock-soil aggregate. Some errors exist for the test results, so only 28 experimental test results of the shear strength of rock-soil aggregate are analysed here. The shear strength of rock-soil aggregate is described by two mechanical parameters, cohesion and friction angle. These experimental tests are divided into two conditions, unsaturated sample and saturated sample. The water content for unsaturated samples is approximately 9.0–13.0%, and the water content for saturated samples is approximately 13.0–18.4%. The degree or saturation for unsaturated samples is approximately 70–80% and 95–98% for saturated samples.  Figure 12 shows the shear strength test results of rock-soil aggregate under different water content conditions.

As shown in Figure 12, the cohesion and friction angle of the rock-soil aggregate obviously decrease with the increasing water content for the unsaturated sample, but for the saturated sample, the change of shear strength is not so obvious. The cohesion and friction angle of the rock-soil aggregate under the saturated condition are generally less than the unsaturated condition, so in the reservoir impounding process, the safety factor of the rock-soil aggregate slope will decrease. The cohesion is more sensitive to water content than the friction angle. The cohesion is 54.3 kPa when the water content is 9.1%, and the cohesion is 18.7 kPa when water content is 18.4%. The decrease ratio is 65.6%. The friction angle is 30.2° (tan 31.8° = 0.582) when water content is 9.1%, and the friction angle is 28.4° (tan 28.4° = 0.541) when water content is 18.4%. The decrease ratio is 7.1%.

The relationship of cohesion, friction angle, and water content of rock-soil aggregate at the Gendakan slope can be described by a fitting equation as follows:

(1) $$\begin{array}{c} y=\frac{a}{1+be^{-cx}}, \end{array}$$

where *y* is the cohesion or friction angle of the rock-soil aggregate; *x* is the water content of the rock-soil aggregate; and *a*, *b*, and *c* are the fitting parameters for the equation. This equation does not really reflect the mechanical relationship of shear strength and water content. Only a fitting function is used to describe how mechanical characteristics varied with the water content of rock-soil aggregate at the Gendakan slope.

The parameters for the fitting equation are as follows.

1. Cohesion in Figure 12(a) is *a* = 17.131, *b* = −3.516, and *c* = 0.179, and the correlation coefficient *R* ^2^ is 0.917.
2. Friction angle in Figure 12(b) is *a* = 28.942, *b* = −58.972, and *c* = 0.733, and the correlation coefficient *R* ^2^ is 0.996.

The stability of the rock-soil aggregate slope is mainly controlled by the bottom layer of rock-soil aggregate, which has a contact surface with bedrock. The water content of the bottom layer of rock-soil aggregate is approximately 8%–10%, so a water content of 9% is selected for natural rock-soil aggregate, and 13% is selected for the saturated rock-soil aggregate under heavy rainfall conditions or behind water level.  Table 4 shows the cohesion and friction angle values of the rock-soil aggregate under natural and saturated conditions for the slope stability analysis. Equation (1) is used to compute the cohesion and friction angle.

As shown in Table 4, the cohesion and friction angle for natural rock-soil aggregates are 57.7 kPa and 31.3°, respectively; the cohesion and friction angle for saturated rock-soil aggregates under heavy rainfall conditions are 26.1 kPa and 29.1°, respectively.

## 5. Stability Analysis of Rock-Soil Aggregate Slope

### 5.1. Deformation and Failure Mechanism

**Figure 13:**
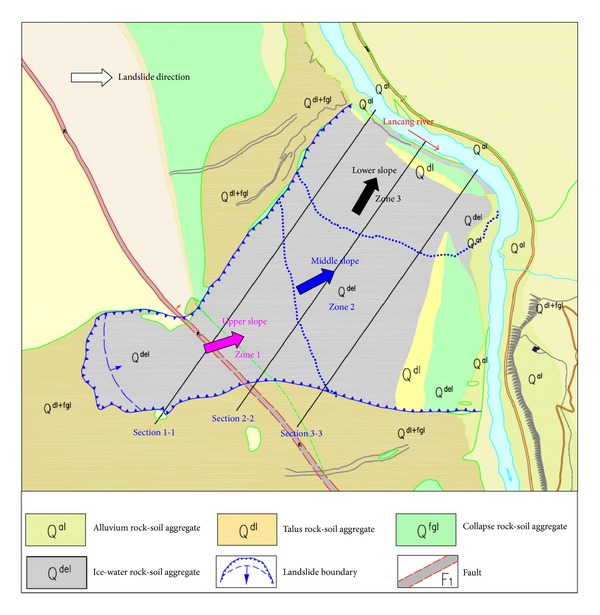
Engineering geological condition of Gendakan slope in plane.

The basic geological characteristics of Gendakan slope are shown in Section 3.1.  Figure 13 shows the engineering geological condition of the Gendakan slope in plane.

**Figure 14:**
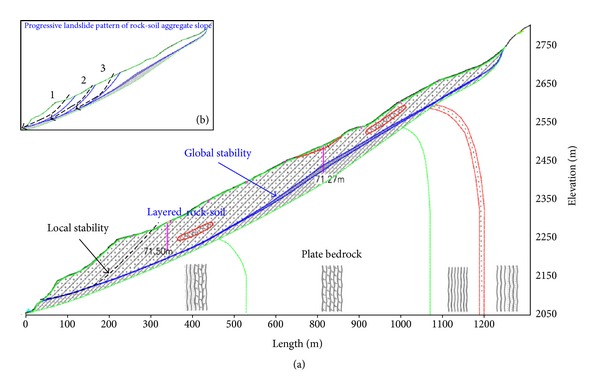
(a) Engineering geological condition of Gendakan slope in Section 1-1 and (b) progressive landslide pattern of rock-soil aggregate slope.

As shown in Figure 13, the volume of the Gendakan slope is enormous. The whole slope can be divided into three zones: upper slope zone (zone 1), middle slope zone (zone 2), and lower slope zone (zone 3). The landslide direction for each slope zone is different, and there is a rotation of the landslide direction for the Gendakan slope. Combined with the engineering geological survey and the mechanical characteristics of rock-soil aggregate, there may be two landslide types for the Gendakan slope: whole slope landslides along the bottom rock-soil aggregate layer and local arc-shaped landslides at the lower slope.  Figure 14(a) shows the engineering geological condition the of Gendakan slope in Section 1-1. 

As shown in Figure 14(a), the global slope stability is dependent on the gravity of the rock-soil aggregate and the shear strength of the bottom rock-soil aggregate layer. Also, the local slope has a free surface, which increases the probability of landslide. The probability of a local arc-shaped landslide is greater than that for a whole slope landslide.  Figure 14(b) shows the progressive landslide pattern of the rock-soil aggregate slope. As shown in Figure 14(b), the local arc-shaped landslide will occur in the lower slope under heavy rainfall conditions. This landslide generates another free surface for the next arc-shaped landslide. The next stage of the arc-shaped landslide will occur at a specific time. The deformation and failure mechanism of the rock-soil aggregate slope is a progressive process, and local landslides will occur step by step.

**Figure 15:**
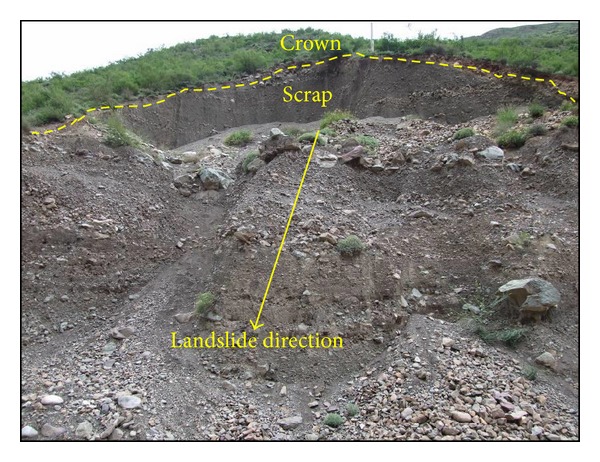
An arc-shaped landslide of rock-soil aggregate slope under rainfall condition in the Gushui Hydropower Station region.

Figure 15 shows an arc-shaped landslide of a rock-soil aggregate slope under rainfall conditions in the Gushui Hydropower Station region.

As shown in Figure 15, the edge of the arc-shaped landslide is clear. This landslide example is a typical landslide form for the rock-soil aggregate slope at the Gushui Hydropower Station region.

### 5.2. Slope Stability Analysis

The three types of slope stability problems for the Gendakan slope, as shown in Figure 13, are global slope stability (zone 1+2+3), local slope stability case 1 (zone 2+3), and local slope stability case 2 (zone 3). In this paper, a three-dimensional limit equilibrium method (3D Bishop Method) is adopted to compute the safety factor of the rock-soil aggregate slope [23]. Because the safety factor in three-dimensional conditions is influenced by the landslide direction, the landslide direction for each slope is a certain value, according to the direction of mesh grid. The lower rock-soil aggregate layer is selected as the bottom sliding surface, and several numerical experiments are carried out to make sure of the dangerous local bottom sliding surface.

**Figure 16:**
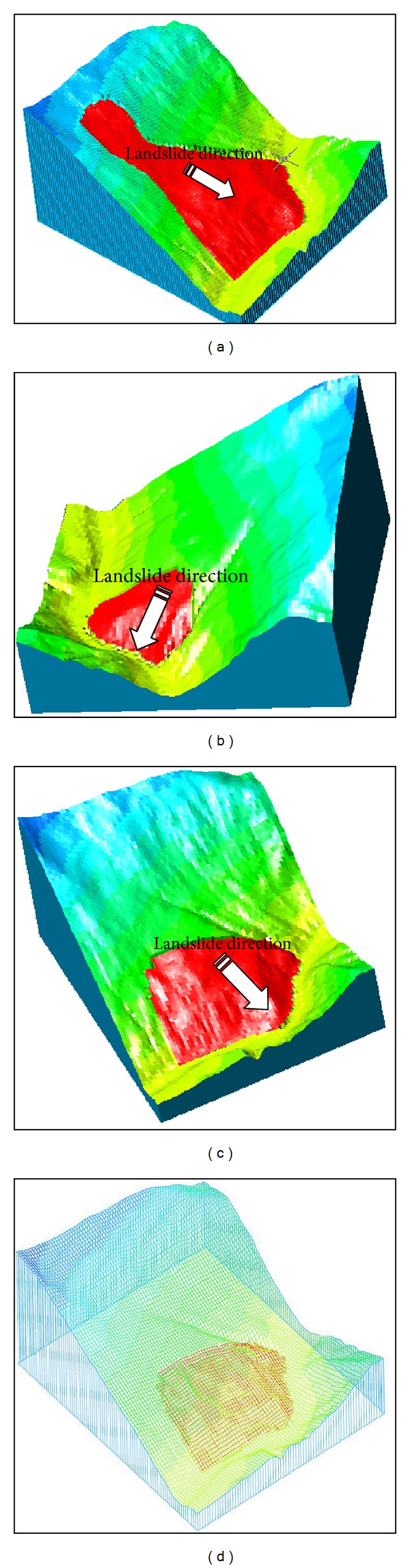
Three-dimensional limit equilibrium computer model under different conditions: (a) global slope stability; (b) local slope stability case 2; (c) local slope stability case 1, and (d) three-dimensional mesh for (c).

**Figure 17:**
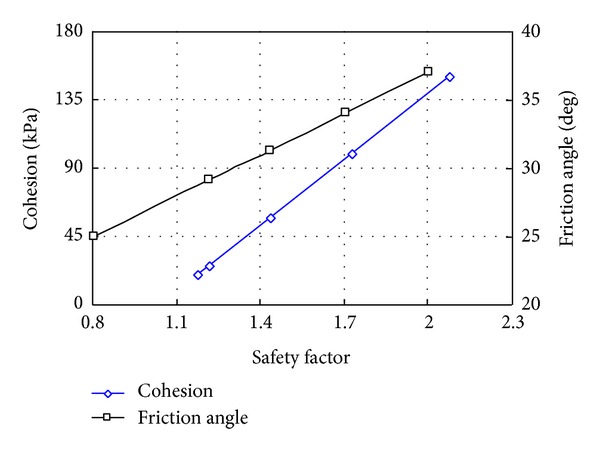
Sensitivity analysis results of shear strength parameters and safety factor.

Two conditions for rock-soil aggregate slope are considered: natural slope and heavy rainfall.  Figure 16 shows the three-dimensional limit equilibrium computer model under different conditions.  Table 5 shows the computed results for the safety factor of the Gendakan slope under different conditions. And a sensitivity analysis of the shear strength parameters impact on the safety factor of slope is carried out based on the global slope stability; Figure 17 shows the sensitivity analysis results of shear strength parameters and safety factor of global slope stability.

As shown in Figure 17, the safety factor of global slope stability is decreased with the decreasing shear strength; the relationship of shear strength and safety factor is a linear function. As shown in Table 5, the safety factor of the whole slope in a natural status is 1.435, and the value under heavy rainfall conditions is 1.215. The safety factor of local slope case 1 in natural status is 1.368, and the value under heavy rainfall conditions is 1.136. The safety factor of local slope case 2 in natural status is 1.159, and the value under heavy rainfall conditions is 0.953. The computed results show that the whole stability of the Gendakan slope is good, but the local stability at the lower slope is bad, especially for zone 3, where an arc-shaped landslide will occur under heavy rainfall conditions. The main reason for a landslide not occurring at the lower rock-soil aggregate slope is the low rainfall conditions at the Gushui Hydropower Station region. 

The stability problem of the rock-soil aggregate slope is the key issue for the safe construction and operation of the Gushui Hydropower Station. The stability of the rock-soil aggregate slope is poor. When some rock-soil aggregate slopes are under water when the reservoir is operational, the stability of the rock-soil aggregate slope will decrease, and a landslide will occur. A very large landslide volume of rock-soil aggregate will impact the safety of the rockfill dam. From the upper stability analysis of the rock-soil aggregate slope, the analysis results show that the landslide probability of whole rock-soil aggregate is small, and the failure of the rock-soil aggregate slope is a progressive process. The local and small volume landslides of rock-soil aggregate impact on the rockfill dam are small. Excavation and support methods for rock-soil aggregate slopes should be performed to ensure the slope stability, but the investment needed for the project will be large.

## 6. Conclusions

In this paper, the Gendakan slope is selected as a case study example for geotechnical characteristics and stability analysis of rock-soil aggregate slope. The glaciers are melting and generating water; large amounts of rock and soil particles at the slope surface are carried by the movement of this water, and they migrated to the lower parts of the slope. The slope evolution can be divided into many stages, so the layered characteristics of the rock-soil aggregate are obvious. The key reasons for the toppling failure of the plate rock masses are the increasing weight of the upper rock-soil aggregate and the mountain erosion by river water. Also, the stress in the shallow plate rock masses is increased. Combined with the increasing stress and decreasing rock mass mechanical parameters, the toppling failure will occur.

The shear strength of the rock-soil aggregate is influenced by the water content and the particle size distribution characteristics. The statistical results for the 30 sets of shear strength of the rock-soil aggregate show that most cohesion values are in the range of 20–60 kPa, and most friction angles are in the range of 24°–36°. The experimental test results show that the cohesion and friction angle of the rock-soil aggregate are decreased by increasing water content. Based on the fitting equation of shear strength parameters, the cohesion and the friction angle for the natural rock-soil aggregate (water content is 9%) are 57.7 kPa and 31.3°, respectively, and for the saturated rock-soil aggregate under heavy rainfall conditions (water content is 13%), are 26.1 kPa and 29.1°, respectively. Combined with engineering geological survey and the mechanical characteristics of rock-soil aggregate, there may be two landslide types for the Gendakan slope: whole slope landslides along the bottom rock-soil aggregate layer and local arc-shaped landslides at the lower slope. The local landslide at the lower rock-soil aggregate slope will occur under heavy rainfall conditions.

## Figures and Tables

**Table 1:** Classification of the toppling failure of plate rock masses.

Type	Dip of rock layer	Geological characteristics	Position
Strong toppling	Angle of toppling rock layer and normal rock layer is larger than 60°.	A clear breakage phenomenon in rock, the continuity of fracture surface is good and extends in a long length, and each surface is distribution of parallel strips in different distances. The crack in fracture zone is mainly opened, no filling of rock block or debris. The phenomenon of shear dislocation is obvious, and several sets of joints are generated by the toppling effect.	(1) Upper part of slope (2) Upper part of plate rock mass (3) Horizontal depth is about 50 m.
Weak toppling	Angle of toppling rock layer and normal rock layer is less than 60°.	The strata dip is abnormal, but the breakage phenomenon is not obvious; the distribution of rock mass is multilayer and continuous. Most of them maintain the organization and structure of original rock mass, but the shear strength is decreased in the local region. The crack in fracture zone is partly opened, and there is a filling of calcite crystals or calcarenite.	(1) Lower part of slope (2) Below the strong toppling rock masses (3) Horizontal depth is about 100 m.

**Table 2:** An example of shear strength test results for plate basalt and joint surface.

Type	No.	Physical characteristic	Peak value		Residual value	
			Frication angle (°)	Cohesion (MPa)	Frication angle (°)	Cohesion (MPa)
Basalt	R-1	Weak weathering	51.12	2.20	43.62	1.61
	R-2	Weak weathering	51.78	2.25	45.00	1.68

Joint	J-1	Rigid	36.87	0.50	35.26	0.30
	J-2	Debris silted	22.29	0.21	21.31	0.17

**Table 3:** Some cohesion and friction angle values for rock-soil aggregate in China [20–22].

Location (position, province)	Cohesion (kPa)	Friction angle (°)	Material characteristics
Xiaowan, Yunnan	50.0	36.0	Mixture of rock block, boulders, and gravel soil; rock content is approximately 32%; rock diameter is 30–350 mm.
Hutiao Valley, Yunnan	12.6	36.5	Mixture of broken stone and rock block; rock content is approximately 46%; rock diameter is 0.1–1.0 m.
Lancang River, Yunnan	48.0	35.0	Mixture of broken stone, rock block, boulders, and silt; rock content is 20%–35%; rock diameter is 0.3–5.0 m, dense structure.
Qingshui River (no. 1), Yunnan	35.0	31.0	Clay cementation of pebble and basalt block.
Qingshui River (no. 2), Yunnan	65.0	30.0	Calcarenite and clay filling of broken stone and sandstone block.
Unknown slope (no. 2), Yunnan	60.0	35.0	Mixture of slate block, limestone block, and clay; rock diameter is 30–80 mm, loose structure.
Liangjiaren, Yunnan	40.0	29.0	An ice-water deposit, mixture of broken stone, boulders, and silt; rock content is 25%–35%.
	18.0	19.0	An ice-water deposit, mixture of broken stone, rock block, and silt; rock content is 25%–35%.
Qianjiangping slope, Hubei	23.0	20.0	Mixture of broken stone, pebble, and clay; the maximum of rock block diameter is 1.5 m; pebble diameter is 30–100 mm.
Baiyiyan, Hubei	47.9	33.8	Calcarenite and sandy soil filling of broken stone.
	55.5	35.4	Mixture of limestone block, sandstone block, and clay.
Huangshi, Hubei	50.0	40.0	Mixture of limestone block, sandstone block, and clay.
	30.0	32.8	Mixture of breccia block, limestone block, and clay.
Yunyang, Chongqing	26.3	13.3	Mixture of sandstone block, broken pebble, and silty clay; rock content is approximately 20%; rock diameter is 2–20 mm.
Fengjie, Chongqing	42.6	26.4	Mixture of broken stone and clay; rock content is approximately 15%; rock diameter is 10–20 mm.
	94.6	28.8	Mixture of breccia block, broken stone, and clay; rock content is approximately 55%; rock diameter is 30–50 mm.
Anle, Chongqing	55.0	32.0	Mixture of broken stone, sandstone block, and sandy clay; rock diameter is 100–800 mm, talus type.
	38.0	25.0	Mixture of pebble and sandy soil; rock diameter is 20–80 mm, alluvium type.
Three Gorges reservoir, Chongqing	25.0	30.0	Mixture of rock block and silty clay.
Dashiban, Sichuan	25.0	13.2	Mixture of rock block and clay; rock content is 25%–35%.
Jinsha River, Sichuan	48.0	39.0	Mixture of limestone block, pebble, and clay; the maximum rock diameter is approximately 4 m.
Feishuiya, Sichuan	60.0	38.6	Mixture of limestone block, sandstone block, and clay.
Xiaoliang Mountain, Sichuan	65.0	31.4	Mixture of pebble and sandy clay.
Dahaizi, Sichuan	36.0	33.0	Mixture of broken stone and sandy soil.
Baishuizhai, Sichuan	37.2	23.1	Mixture of limestone block, phyllite block, and clay.
	46.5	28.0	Mixture of limestone block and sandy soil, dense structure.
Jinsha reservoir, Sichuan	30.0	17.0	Mixture of broken stone, sandstone block, and silty clay.
Yalong River, Sichuan	60.0	35.0	Mixture of broken stone, sandstone block, and clay; rock content is approximately 30%; rock diameter is 400–1000 mm.
Shiwan, Sichuan	10.0	25.4	Mixture of pebble, granite block, and clay; rock diameter is 40–150 mm.
Unknown slope, Jiangxi	10.0	25.0	Mixture of rock block and clay.

**Table 4:** Cohesion and friction angle values of rock-soil aggregate under natural and saturated conditions for the slope stability analysis.

Condition	Water content (%)	Shear strength	
		Cohesion (kPa)	Friction angle (°)
Unsaturated (natural condition)	9	57.7	31.3
Saturated (heavy rainfall)	13	26.1	29.1

**Table 5:** Computed results for the safety factor of Gendakan slope under different conditions.

Conditions	Natural slope	Heavy rainfall
Global slope stability	1.435	1.215
Local slope stability case 1	1.368	1.136
Local slope stability case 2	1.159	0.953
